# Supplementary material for: Pheromone Activity after Stimulation with Ampicillin in a Plasmid-Free Enterococcus faecalis Strain
Source: Microorganisms. 2022 Nov 19;10(11):2294. doi: 10.3390/microorganisms10112294 (PMC9692682; doi:10.3390/microorganisms10112294)
Supplement: Supplementary file 1 [file microorganisms-10-02294-s001.zip › microorganisms-2020388-supplementary.pdf]

microorganisms

Supplementary figures

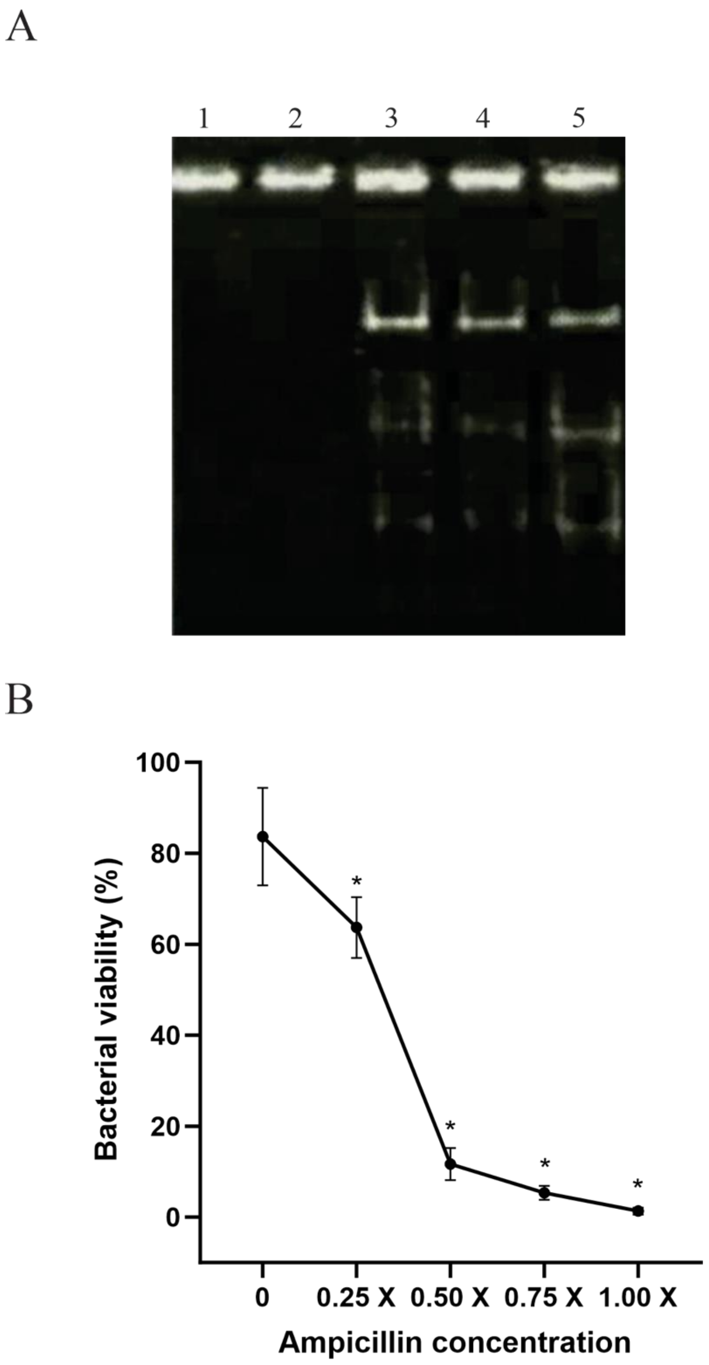

Supplementary figure S1. Induction of a native plasmid with markers of resistance and selection of resistance with a sub-inhibitory stimulus. (**A**) The clinical strain 1390 was stimulated with sub-inhibitory concentrations of ampicillin from 0.25X MIC (0.5 µg/ml) to 1X MIC (2 µg/mL). Line 1. 1390 without stimulus, 2. 1390 0.25X, 3. 1390 0.5X, 4. 1390 0.75X and 5 1390 1X. Each culture was grown for 24 h; the cells were then harvested, and the plasmid profile was obtained. The figure shows that plasmid could be isolated until after the antibiotic stimulus, when the selection of clones without plasmid became eviden ($P<0.01$). (**B**) Follow-up of bacterial viability during the treatment and under selective pressure. A loss of viability was obvious in all treatments; however, a small percentage of bacterial cells were able to survive (1% of

population) even when the antibiotic stimulus reached 1X MIC. Statistical significance is indicated by a single asterisk (*) for p = < 0.05.

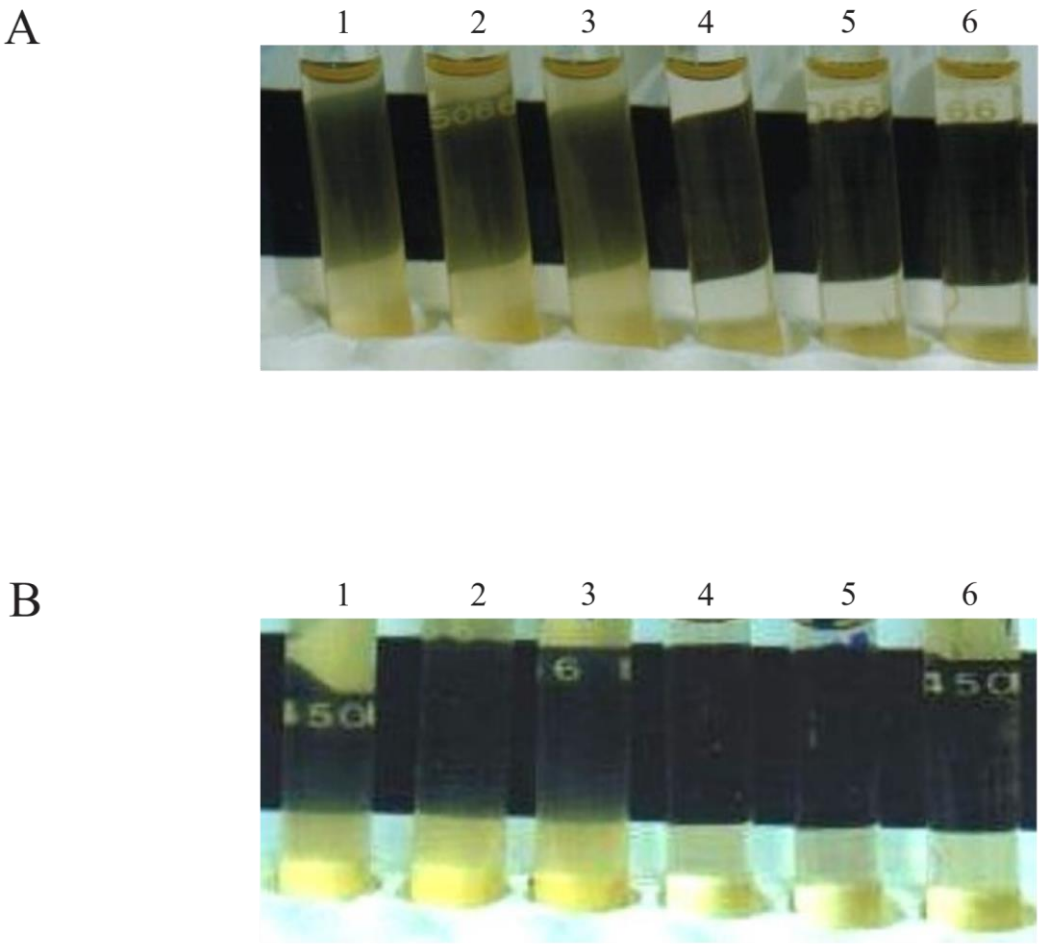

Supplementary figure S2. Minimal inhibitory concentration. (**A**) The wild-type 1390 strain exhibited a minimal inhibitory concentration of 2 µg/ml (1. 0.5, 2. 0.75, 3.1, 4. 2, 5, 4, 6, 8 µg/mL). (**B**) Minimal inhibitory concentration after continuous sub-inhibitory stimulus with ampicillin. The MIC increased up to 128 µg/mL, and a resistant clone emerged (1.16, 2. 32, 3 64, 4. 128, 5. 256, 6. 512 µg/mL).

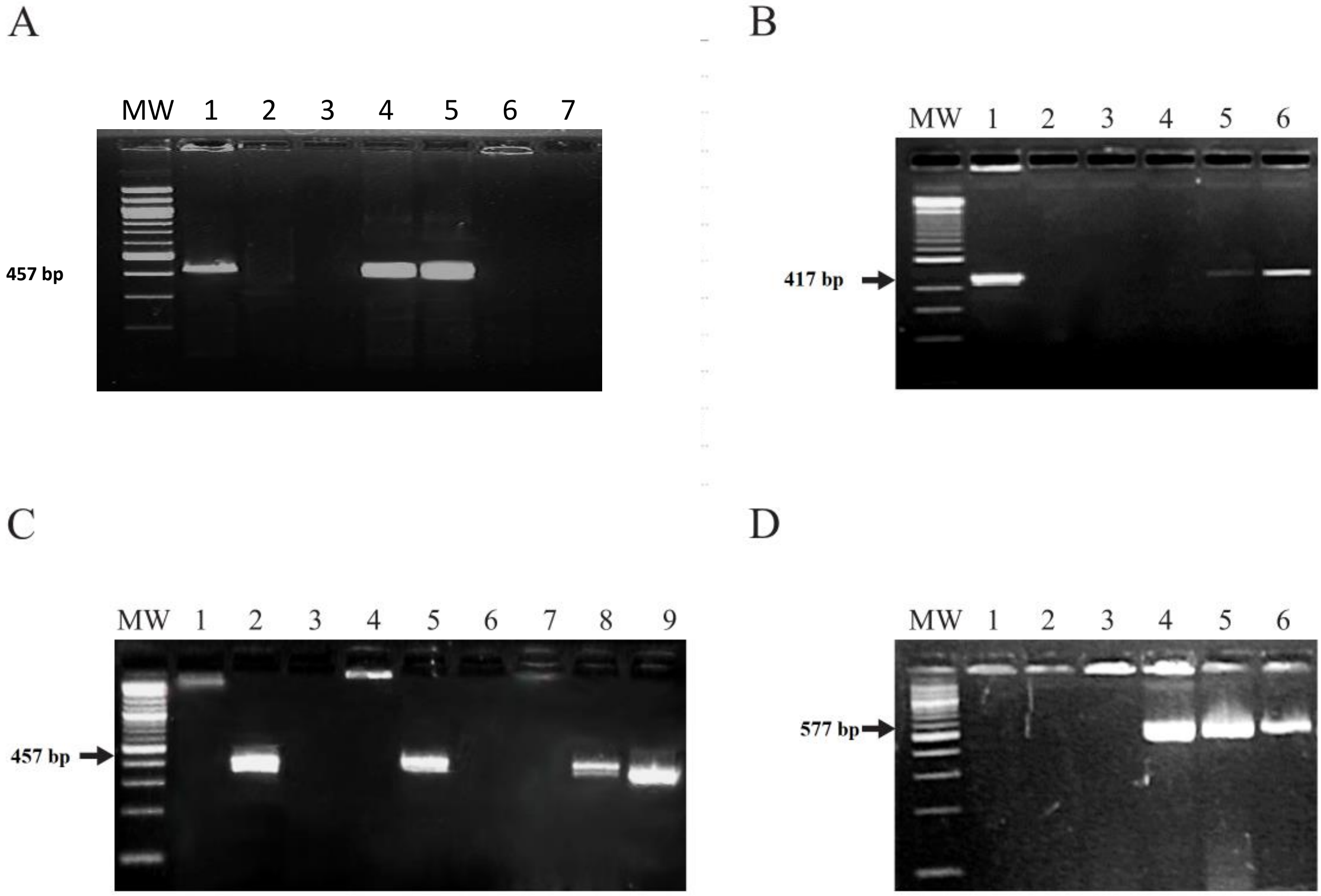

Supplementary figure S3. Analysis of the effect of curing with 10 mM ascorbic acid on the plasmid markers *iad1* and *prgB*. A) Expression of *prg* B of 1390R stimulated with supernatants from C29, MW molecular weight marker, 1. *E. faecalis* ATCC 29212 (ampicillin-sensitive reference strain), 2. *E. faecalis* C29, 3. *E. faecalis* C29 0.5X MIC, 4. 1390R without stimulus, 5. 1390R stimulated with supernatant from C-29 with 0.25X of MIC of ampicillin, 6. 1390R stimulated with supernatant from C-29 with 0.5X of MIC of ampicillin and 1390R stimulated with supernatant from C-29 with 0.5X of MIC of ampicillin. B) Expression of *iad1* in C29 (absence) and 1390R (presence), MW. Molecular weight marker, 1. *E. faecalis* ATCC 51299 (vancomycin-resistant control strain), 3. C29 0.5X, 4. C29, 5. 1390S, 6. 1390R. C) The *prgB* gene encodes aggregation substances on the plasmid. MW. Molecular weight marker, 1. *S. aureus* 29213, 2. *E. faecalis* ATCC 29212 (ampicillin-sensitive reference strain), 3. *E. faecalis* C29, 4. *E. faecalis* C29 0.5X MIC, 5. plasmid from 1390R, 6. purified DNA from 1390R, 7. purified DNA from C29, 8. 1390S, 9. 1390R. D) Presence of phosphotransferase *ant(6)-I*, which is responsible for HLSR. MW. Molecular weight marker 100 bp, 1. *E. faecalis* ATCC 29212, 2. *S. aureus* 29213, 3. C29, 4. *E. faecalis* 51299, 5. 1390 2X MIC, 6. 1390R.
